# Notes and Miscellany

**Published:** 1885-05

**Authors:** 


### NOTES AND MISCELLANY.

"Who left that door open?" growled Mr. Dinkle, looking up from his desk one of those freezer days last week. "I did," answered the new office boy. "Can't you ever learn to shut a door?" "I s'pose so, sir." "Well, why in thunder don't you do it?" "I'm goin' to, but you see I'm new yet, and I had so much to learn that I thought I'd leave the door be till along towards the last.—*Merchant Traveler.*

=A Cure for Hiccough.—A remedy, tested many times without a failure, is published in the *Popular Science Monthly*, which says that it can always be used by some one else upon a person who has "the hiccoughs," and generally by the sufferer himself. You say to your friend something like this: "See how close together you can hold the tips of your forefingers without touching. Now keep your elbows out free from your side. You can get your fingers closer than that. They are touching now. There—now hold them so. Steady!" By this time you can generally ask: "Now why don't you hiccough?" The involuntary tendency to breathe slowly and steadily when the attention is fixed on performing a delicate manipulation counteracts the convulsive action of the diaphragm.

=Language, you know, was given to man to conceal his thoughts, and you never realize it so intensely as you do when you see the minister sit down violently just after his heel has found a square inch or so of ice under the snow, and hear him mildly ejaculate, "Dear me!" —*Somerville Journal.*

=A recent German publication contains a description of a new electric plant that has been christened Phytolacca electrica, which possesses stronly marked electro-magnetic properties. In breaking a twig the hand receives a shock that resembles the sensation produced by an induction coil. Experiments made on this plant with a small compass showed that the compass was affected by it at the distance of about twenty feet. On a nearer approach the needle vibrated and finally began to revolve quite rapidly. The phenomena was repeated in reverse order on receding from the plant. The energy of the influence varied with the time of day—being strongest at about 2 o'clock P. M., and becoming almost nothing during the night. It was also greatly increased in stormy weather; and when it rains the plant seems to wither. It is said that no birds or insects are ever seen on or about this plant. The soil where it grew contained no magnetic metal, like iron, cobalt, or nickel, and it is evident the plant itself possessed this electrical property.—*Gardener's Monthly.*

"You look dreadfully tired," said the sleigh to the wheel. "That's because I go 'round with the felloes, I suppose," said the wheel. "I get awfully slewed myself, sometimes," remarked the sleigh." "I am always pretty full when I go to a funeral," said the carriage, sticking out its tongue. Then the wheel spoke again and said, "Stop the hubbub! Here is a couple of awful cross roads ahead."—*Boston Commercial Bulletin.*

---

=Alcohol and the Voice.—Dr. Lennox Browne, before the Society for the Study and Cure of Inebriety, said that out of nearly 400 vocalists, 101 were total abstainers, and many of these were the chief cathedral singers, 65 only took stimulants at meals, 65 only took them at supper, 47 both at meals and supper, while the remaining took stimulants whenever they felt inclined and an opportunity offered ; 75 per cent. never took stimulants immediately before singing, while 20 per cent. occasionally did so. Arguing from these figures, Dr. B. was of opinion that alcohol is by no means necessary ; in fact, the habitual use of it only tended to render the voice husky and indistinct.—*Sanitary Engineer.*

---

=The bathrooms of Japanese hotels are conducted in a manner that has the charm of novelty to American tourists. The bath-tub and heater are combined so that the water, once heated, must furnish the bathing material for the whole house. Arriving at a Japanese hotel footsore and weary, you ask the landlady : "How many have used the bath?" She innocently replies, "Only eight." You forego the luxury of such a bath.—*Hotel Mail.*

---

=The miserable young woman in the next house to me spends all her young, bright days, not in learning to darn stockings, sew shirts, bake pastry, or any art, mystery or business that will profit herself or others ; not even in amusing herself or skipping on the grass plots with laughter of her mates ; but simply and solely in raging from dawn to dark, to night and midnight, on a hapless piano, which it is evident she will never in the world render more musical than a pair of barn-clappers. The miserable young female! Carlyle.

---

=A clergyman at Cambridge preached a sermon which one of his auditors commended. "Yes," said the gentleman to whom it was mentioned, "it was a good sermon, but he stole it." This was told to the preacher. He resented it, and called on the gentleman to retract what he had said. "I am not," replied the aggressor, "very apt to retract my words, but in this instance I will. I said you had stolen the sermon ; I find I was wrong, for on my return home, and referring to the book whence I thought it was taken, I found it there."—*Harper's Monthly.*

